# High‐capacity protein A affinity chromatography for the fast quantification of antibodies: Two‐wavelength detection expands linear range

**DOI:** 10.1002/jssc.201701481

**Published:** 2018-02-07

**Authors:** Peter Satzer, Alois Jungbauer

**Affiliations:** ^1^ Department of Biotechnology University of Natural Resources and Life Sciences Vienna Austria; ^2^ Austrian Centre of Industrial Biotechnology (ACIB) Vienna Austria

**Keywords:** antibodies, hexameric protein A, high‐throughput analysis, immunoglobulin, quantification

## Abstract

The high‐throughput analysis of antibodies from processes can be enhanced when the linear range is expanded and sample preparation is kept to a minimum. We developed a fast chromatography method based on a hexameric variant of staphylococcal protein A immobilized on Toyopearl matrix, TSK 5 PW using two wavelengths. A protocol with 5 min runtime and a single‐wavelength detection at 280 nm yielded an upper limit of quantification of 2.10 mg/mL and a lower limit of quantification of 0.06 mg/mL. The optimized method with a runtime of 2 min and two‐wavelength detection at 280 and 300 nm allowed us to span a valid concentration range of 0.01–5.20 mg/mL using two calibration curves. Sample selectivity was tested using mock supernatant mixed with antibody concentrations of 0.1–2.1 mg/mL, sample stability in the autosampler was shown for at least 24 h. We also tested the capabilities of the method to determine purity of an antibody sample by calculating the ratio of peak area of elution to peak area of flow‐through, which correlated well with the expected purity. The method will be very useful for process development and in‐process control, spanning concentrations from seed fermentation to harvest and purification.

**Abbreviation**
HCP: host cell proteins

## 1 INTRODUCTION

Antibody analysis using staphylococcal protein A is frequently applied for process development, in‐process control and also final control in antibody production. The methods have to be robust, fast, and minimal sample pretreatment is desired. Recently novel staphylococcal protein A variants have been developed, one is the hexameric ligand derived from the mutated C‐domain. The binding affinity and the equilibrium capacity of this ligand are very high. It is very well established that with ligand density and affinity the linear detection range can be increased as long as the response of the detector is also linear. Protein A affinity ligands have been already used for analysis of antibodies from culture supernatant and process samples, but cannot span the whole range from seed fermentation to final product without sample treatment. The analytical potential of protein A affinity chromatography has been realized at the same time when protein A affinity chromatography was progressively used for preparative and industrial antibody purification. Lacking chromatography material design for analytics, with small beads and high pressure stability initially the chromatography material designed for industrial scale purification was also used for analysis of antibodies 1. Due to the potential of antibody analytics, several different formats besides traditional chromatography material using staphylococcal protein A ligands have been successfully developed. Protein A ligands have been immobilized on capillaries filled with monoliths 2, monolithic discs 3, 4 wide pore material such as Poros used for perfusion chromatography 5, 6, dextran grafted agarose media 7 and capillary‐channeled polymerpolypropylene fibers 8, 9. Also conjoint LC originally proposed by Tennikova 10 has been used to determine antibodies and other proteins in a single step 11. Protein A affinity columns are also used for at‐line monitoring 5 and in tandem with SEC to determine the quantity of antibodies and the aggregate content 12, 13, 14. Common to these methods is the limited concentration range, although the methods have been gradually improved over time. To detect a wide range and to minimize sample dilution, the elution can be monitored by more than one wavelength. At low concentration a more sensitive wavelength is used in contrast to high concentrations, where a less sensitive wavelength can be used. Antibodies have a maximum in UV absorption at 280 nm. For high concentrations, 300 nm are less sensitive and the detector is still in the linear range. Currently, the procedure for characterization of an analytical method is very well established and we used methodology described by Hartmann et al. 15. The limits of detection and quantification are determined as well as the inter‐ and intra‐day variation. The methods proposed by different authoritative bodies are very similar and only vary in minute detail. To assess the specificity, the best method is to use a mock cell culture supernatant, which can be spiked to the antibody to simulate different concentrations and mixing rations between host cell proteins (HCPs) and antibodies. The unspecific adsorption, which is the main cause for the lower LOD, can be monitored by comparison with a pure mock injection. In this work, we have used an analytical HPLC column with a new hexameric ligand, which is a mutated staphylococcal protein A variant with a high affinity and equilibrium capacity. The method was successfully validated for two different wavelengths to expand the calibration range of the method. Lower LOD and quantification were determined along with inter‐ and intra‐day precision and selectivity by using mock cell culture.

## 2 MATERIALS AND METHODS

All chemicals were of analytical grade and purchased from Sigma–Aldrich, unless stated otherwise.

### 2.1 UV 280 measurement and concentration determination

For UV measurement a Cary 60 UV‐vis (Agilent) was used. Protein concentration of pure antibody was measured at 280 nm and the antibody concentration was calculated using the molar extinction coefficient.

### 2.2 TSK Tosoh protein A chromatography

Analytical protein A chromatography was performed using a TSK 5PW protein A column (Tosoh) with an inner diameter of 4.6 mm and a length of 3.5 cm. The column was connected to a Dionex U3000 (Thermo Fisher) equipped with a Dionex Ultimate 3000 DAD detector (10 mm pathlength) and a Dionex WPS‐3000 TSL Micro Autosampler. The column was equilibrated (30 mM potassium phosphate, pH 7.5, 150 mM NaCl) at 2 mL/min and 10 or 50 μL of filtered (0.2 μm, Millipore) sample was injected. After a wash step, the bound protein was eluted using 0.01 M HCl. The UV absorbance was monitored at 280 and 300 nm. For antibody peak determination, the 280 or 300 nm antibody peak was integrated and compared with a calibration curve to calculate mAb concentration. For purity determination, the flow through signal at 280 nm and the antibody peak were used to calculate the percentage of signal corresponding to the mAb.

## 3 RESULTS AND DISCUSSION

**Figure 1:**
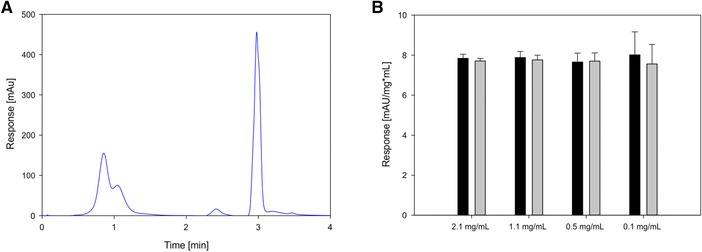
Panel A shows a typical chromatogram of protein A analytical separation of antibody in CHO supernatant. The first peak at 1 min is the HCP flow through and the peak at 3 min is the antibody peak. Panel B shows a comparison between the response normalized by the concentration with the pure antibody diluted in buffer (black bars) and the antibody diluted in mock‐CHO‐supernatant (gray bars), all samples were done in triplicates

The TSK Tosoh protein A column is suitable for fast concentration determination of antibodies. A small column volume and high flow rates reduce the necessary sample time in comparison to other methods, while a high capacity of the ligand ensured high column capacities. The antibody standard used for the validation is an IgG2, kindly provided by LEK, a Sandoz company (Menges, Slovenia) with a molar extinction coefficient of 1.4 and was purified by protein A chromatography. The antibody concentration was measured by UV280 and concentration was calculated using the molar extinction coefficient. The buffers used for chromatography were intentionally generic and simple, a 30 mM phosphate buffer with 150 mM salt at pH 7.5 was used for equilibration, and 0.01 M HCl was used for elution. The column was operated at a flow rate of 2 mL/min (recommended by the manufacturer) and a scheme of 1 min equilibration, 1 min sample load, 1 min wash, 1 min elution and 1 min equilibration was used for a first impression on the capabilities of the method and resulted in a UV280 trace as presented in Figure 1 panel A. The chromatogram shows one initial flow through peak consisting of HCPs (at min 1) and a second peak for the elution of the antibody (at min 3). We considered the small peak at min 2.5 to not be antibody, because this peak was not visible when the method was performed with pure antibody. The tailing of the peak at min 3 was included as antibody, as this tailing was also visible for pure antibody. The antibody peak area was calculated by using two fixed points before and after the antibody peak as baseline points. We performed these experiments with antibody diluted in mock supernatant and diluted in equilibration buffer to prove the selectivity of the method. Differences between the two could not be observed throughout the concentration range we used for the calibration (Figure 1 panel B) proving the selectivity of the method.

### 3.1 Linearity

**Figure 2:**
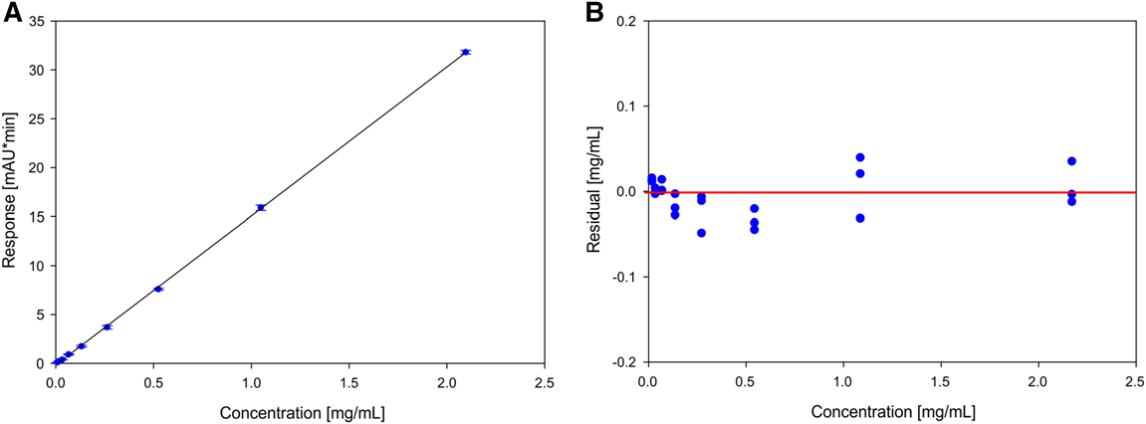
Panel A shows the response of the area of the antibody peak for different concentrations for the complete calibration range with the 95% confidence interval and SDs. All samples were measured in triplicates. Panel B shows the distribution of the residuals for the complete concentration range

To confirm the linearity of the calibration model and to find any issues with saturation of the detector or other non‐linear effects, we tested eight different monoclonal antibody concentrations ranging from 0.016 to 2.1 mg/mL in triplicate. The linearity of the model can be seen in Figure 2 panel A and shows a highly linear response. The linearity was also confirmed using an ANOVA test (linearity *F*‐test, F* = 39694) as well as by inspecting the residuals (Figure 2 panel B). Both the ANOVA and the residuals confirm the linearity over the whole investigated concentration range.

### 3.2 Accuracy and precision

For both accuracy and precision samples of all eight different concentrations were made on eight consecutive days in duplicate. Accuracy and precision were calculated for all eight different concentrations and are presented in Table 1. Criteria for the validity of the concentration range were chosen to be a 20% RSD for the LLOQ (lower limit of quantification) and 15% for the rest of the concentration range, and 20% bias for the LLOQ and 15% bias for the rest of the concentration range. The bias was calculated as deviation from the theoretical value from off‐line UV‐concentration determination using the molar extinction coefficient and the dilution of the sample. Both bias and RSD are within the limits for a concentration range of 66 μg/mL to 2.1 mg/mL, but are out of these limits below 66 μg/mL.

**Table 1:** Precisions and bias for duplicate samples of eight different concentrations measured on eight consecutive days

Concentration (mg/mL)	RSD (%)	Bias (%)
2.096	2.3	−0.2
1.048	3.5	−0.5
0.524	5.6	3.4
0.262	9.4	4.4
0.131	12.8	6.9
0.066	12.8	−3.8
0.033	30.7	0.2
0.016	42.8	−28.6

### 3.3 Stability

**Figure 3:**
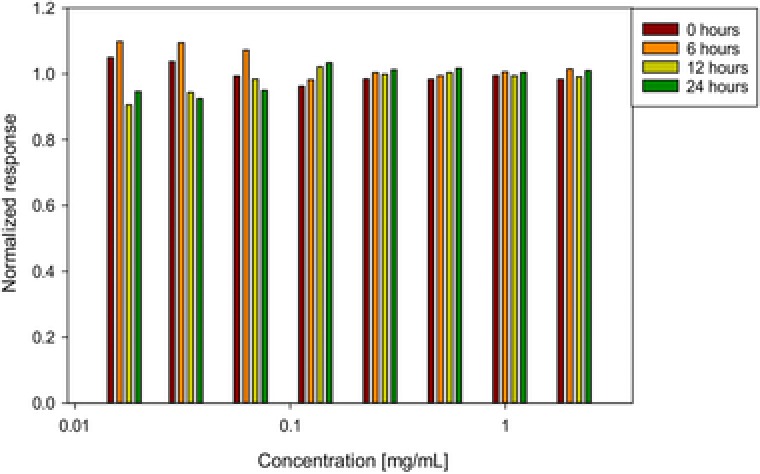
Measurements of different concentrations immediately after preparation and after 6, 12 and 24 h of storage in the autosampler of the HPLC

To test the stability of the samples while being in the autosampler, we tested the same samples in duplicates while being measured immediately after they are put into the instrument, 6, 12, and 24 h later. The autosampler was set to 4°C for the whole duration. The detector response is shown in Figure 3. There was no significant change in signal response detectable in this 24 h. We did not test for longer storage times in the autosampler, because the autosampler of the instrument can hold at maximum three 96‐well plates, and 300 samples can be measured in about 24 h.

### 3.4 Optimization of the method

**Figure 4:**
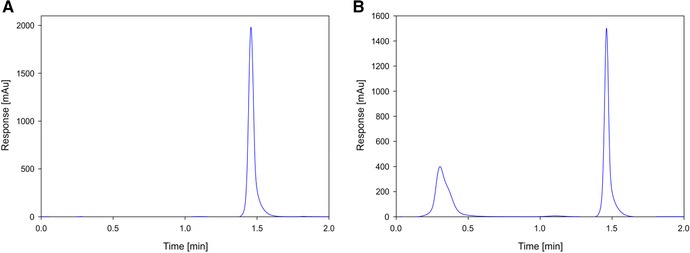
Panel A shows 50 μL injection of 5.2 mg/mL antibody solution, showing no flow through and Panel B shows the chromatogram of CHO supernatant showing the separation of flow through (HCP) and antibody

After validation, we tried to optimize the method in terms of speed, lower limit of quantification, and calibration range. The method was shortened to a 2 min protocol instead of a 5 min protocol, shortening wash and equilibration times as well as the elution to 0.4 min load and wash, 0.6 min elution and 1 min re‐equilibration. The samples were prepared in a 96‐well plate and the injection volume was increased from 10 to 50 μL to boost sensitivity. Because of the promising first results, the concentration range was also increased to 5.2 mg/mL as the highest concentration, down to 3 μg/mL as lowest concentration. There was no flow through detectable for a pure antibody, which indicates that the column is not overloaded even on the highest concentration (Figure 4 panel A). The chromatogram of unpurified supernatant is presented in Figure 4 panel B and shows that flow through and antibody peak are still completely separated despite the shorter method.

### 3.5 Linearity of the optimized conditions

**Figure 5:**
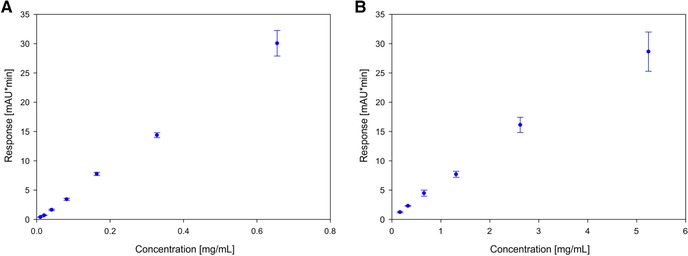
Panel A shows the calibration for 2 min protein A chromatography with 50 μL injection volume in the linear range of the UV280 absorption signal and Panel B shows the data from the same run for UV300 absorption signal and extended linear range up to 5 mg/mL

Linearity was also tested for the optimized conditions with higher injection volume and shorter method duration. We found that at higher concentrations, we see saturation on the detector of the instrument and flattening of the calibration curve (data not shown). We therefore shortened the calibration to 0.65 mg/mL instead of 5.2 mg/mL for the 280 nm signal (Figure 5 panel A), but evaluated the runs for 300 nm absorption as well for the full range up to 5.2 mg/mL (Figure 5 panel B). The 300 nm signal shows linearity up to 5.2 mg/mL concentration and can be used to extend the capabilities of the method, without the need for additional samples or additional dilution.

The increase of the sample volume led to a decrease of the LOQ by a factor of 5, up to a concentration of 10 μg/mL. For lower concentrations, the bias gets over the cutoff limit of 20% bias (Table 2). The true concentrations for calculating bias were calculated based on the offline UV280 measurement of the highest concentration and calculated by using the molar extinction coefficient. The table shows the valid concentration range, and RSD and bias are for the already recalculated concentration range based on LOQ and bias cutoffs of 15% for the whole concentration range and 20% for the LOQ. For 300 nm detection, the concentration range could be extended for higher concentrations of up to 5.2 mg/mL, but with a loss of sensitivity for low concentrations and the concentration range was only valid up to 160 μg/mL. With this two‐wavelength setup for the evaluation of the same sample, concentrations between 10 μg/mL and 5.2 mg/mL can be measured without additional sample preparation or changes in the method which spans almost three orders of magnitude. With this extended protocol for protein A chromatography, we are now able to measure all relevant concentrations in a typical antibody fermentation from the seed fermentation right up to the final harvest in fed batch. Concentrations higher than 5.2 mg/mL have not been routinely achieved yet in commercial antibody fermentations, but in principle this method could maybe still be extended further above 5.2 mg/mL, either using the 300 nm detection signal or even going to higher wavelengths. On the basis of the determined RSD and bias, we recommend using 280 nm as long as it is valid (up to 650 μg/mL) and use 300 nm above that limit.

**Table 2:** Precisions and bias for duplicate samples of eight different concentrations measured on eight consecutive days for optimized 2 min runs with 50 μL injection at two different wavelengths (280 and 300 nm). Bias is shown according to a regression based on the valid concentration range

Concentration (mg/mL)	280 nm RSD (%)	280 nm bias (%)	300 nm RSD (%)	300 nm bias (%)
5.291	6.4	>15	11.7	−3.3
2.621	8.2	>15	8.1	7.1
1.310	6.6	>15	6.6	−0.7
0.655	7.3	0.6	11.6	9.1
0.328	2.9	−3.5	3.2	0.6
0.164	3.6	4.9	7.9	−19.3
0.082	5.2	−5.2	10.2	>20
0.041	7.6	−6.4	7.8	>20
0.020	5.9	−13.6	8.1	>20
0.010	4.5	11.3	4.3	>20
0.005	1.9	>20	11.7	>20
0.003	3.7	>20	8.1	>20

This optimized method now offers great opportunities for very fast and accurate measurement of whole downstream and upstream processes without sample preparations or dilutions for a wide range of different concentrations. For antibodies with IgG1 subtype, we expect even a wider calibration range. Protein A affinity columns have higher binding capacity for human IgG1 than the IgG2 subtype used in this study. Methods developed in the past which offer a wide valid concentration range have to adjust the method, such as adjusting the sample volume. For example, protein A CIM‐DISC chromatography which had a calibration range from 0.02 to 0.25 mg/mL without changing the method has a significantly smaller calibration range, especially for higher concentrations. In this case, the calibration range could only be expanded by changing the sample volume 3. Another method based on protein‐G immobilized on a POROS column for IgG quantification 16 achieved linearity between 0.04 and 0.20 mg/mL with a method runtime of 14 min, which is a significantly smaller range and significantly longer method and lacks both on the lower and upper limit of quantification in comparison to our method. The big difference between these methods and our method is a combination of high binding capacity resin to avoid column overload and the measurement on different wavelength to avoid detector saturation during elution.

### 3.6 Purity

**Figure 6:**
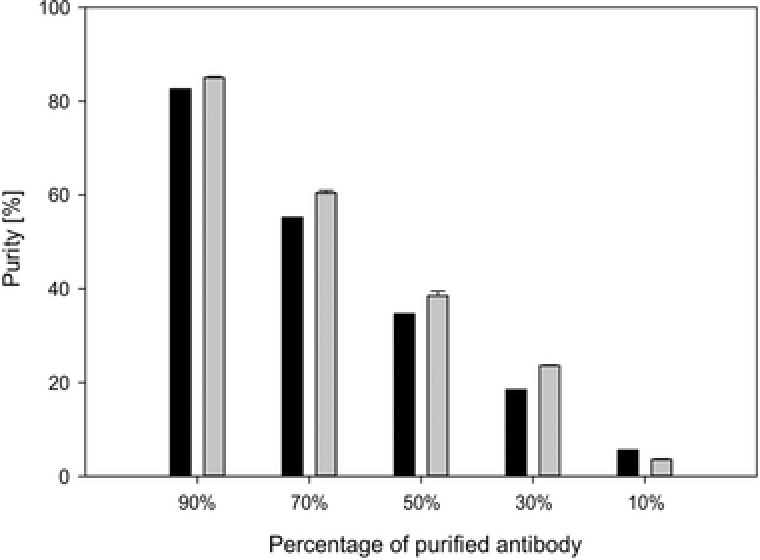
Theoretical purity (in black) and measured purity (in grey) of antibody diluted in mock‐supernatant

Determination of purity and aggregate content can be done either using non‐chromatographic methods like ELISA or chromatography methods such as SEC. While SEC provides high resolution of individual compounds and can also be used for aggregate measurement, it is very time consuming. As we wanted to get as much information from one analytical run we used our method and the relation between the flow through signal and antibody signal to see if an estimation of purity is possible or not. For sample preparation of different purities, different ratios of mock supernatant and purified supernatant were used. For both, the UV280 absorbance was determined before and both were mixed with different mixing ratios (10, 30, 50, 70, and 90% of purified antibody in mock supernatant), which results in purities roughly between 5 and 85%. This is the range which is typically encountered during the fermentation and at the end of fermentation, or the first downstream unit operations. To test the capabilities of this estimation, we calculated a theoretical purity based on the offline measured UV280 absorbance and the mixing ratios and compared it to the purities determined by the area of the flow‐through peak and the antibody peak in the chromatogram. The calculated values were in good agreement with the theoretical purities (Figure 6) but overestimated the purity in all mixing conditions but the last one. It is very doubtful that this measurement can be accurately validated, but it can be used as a guidance for fast and quick estimation during the fermentation from seed fermentation to final harvest, or for the first downstream process steps. The wide calibration range, especially into high antibody concentrations, made this possible without additional sample preparation or instrumentation while at the same time the concentration determination is done.

## 4 CONCLUDING REMARKS

We developed and validated a 2 min analytical protein A method for fast and robust determination of antibody content in complex and purified samples. We were able to validate a very wide quantification range of 10 μg/mL to 5.2 mg/mL using two different wavelengths and a high‐binding‐capacity resin to reduce or eliminate the need for sample preparation and facilitate fast and easy antibody concentration determination. This method is especially interesting either during fermentation to guide the fermentation as an at‐line measurement or as fast offline‐method with minimal sample adjustment. Additionally, we demonstrated that purity estimation is also possible with the same chromatogram without additional work, which will facilitate the current trend of integrated upstream and downstream development. Getting concentration and purity information in a convenient and fast way might enable the upstream to change the paradigm of “high titer is better” to a more complex paradigm of “high titer and purity is better” to lower the cost and burden for downstream.
